# Mechanochemical synthesis and crystal structure of a 1:2 co-crystal of 1,3,6,8-tetra­aza­tri­cyclo[4.3.1.1^3,8^]undecane (TATU) and 4-chloro-3,5-dimethyl­phenol

**DOI:** 10.1107/S2056989016016650

**Published:** 2016-10-25

**Authors:** Augusto Rivera, Jicli José Rojas, John Sadat-Bernal, Jaime Ríos-Motta, Michael Bolte

**Affiliations:** a Universidad Nacional de Colombia, Sede Bogotá, Facultad de Ciencias, Departamento de Química, Cra 30 No. 45-03, Bogotá, Código Postal 110911, Colombia; b Institut für Anorganische Chemie, J. W. Goethe-Universität Frankfurt, Max-von Laue-Str. 7, 60438 Frankfurt/Main, Germany

**Keywords:** crystal structure, mol­ecular co-crystal, mechanochemical synthesis, π–π stacking

## Abstract

Solvent-free treatment of 1,3,6,8-tetra­aza­tri­cyclo­[4.3.1.1^3,8^]undecano (TATU) with 4-chloro-3,5-di­methyl­phenol led to the formation of the title co-crystal, C_7_H_14_N_4_·2C_8_H_9_ClO. The asymmetric unit contains one aminal cage mol­ecule and two phenol mol­ecules linked *via* two O—H⋯N hydrogen bonds. In the aminal cage, the N–CH_2_–CH_2_–N unit is slightly distorted from a *syn* periplanar geometry. Aromatic π–π stacking between the benzene rings from two different neighbouring phenol mol­ecules [centroid–centroid distance = 4.0570 (11) Å] consolidates the crystal packing.

## Abstract 2

In the crystal, the 1:2 co-crystalline adducts are linked by π–π stacking inter­actions.

## Chemical context   

Phenols and cyclic aminals are known to form a variety of supra­molecular aggregates *via* O—H⋯N hydrogen bonds, and complexes of phenols with various nitro­gen bases are model systems often applied in the study of the nature of the hydrogen bond (Majerz *et al.* 2007 ▸). Previously, hydrogen bonding between the hydroxyl group of acidic groups such as phenols and heterocyclic nitro­gen atoms has proved to be a useful and powerful organizing force for the formation of supra­molecules (Jin *et al.*, 2014 ▸). In a continuation of our previously published work in this area (Rivera *et al.*, 2007 ▸, 2015 ▸) and as a part of our research on compounds in which a cyclic aminal acts as a central host and organizes guest mol­ecules around it *via* hydrogen bonding, we report herein the synthesis and crystal structure of title compound. This was assembled through hydrogen-bonding inter­actions between the cyclic aminal 1,3,6,8-tetra­aza­tri­cyclo­[4.3.1.1^3,8^]undecane (TATU) and 4-chloro-3,5-di­methyl­phenol.
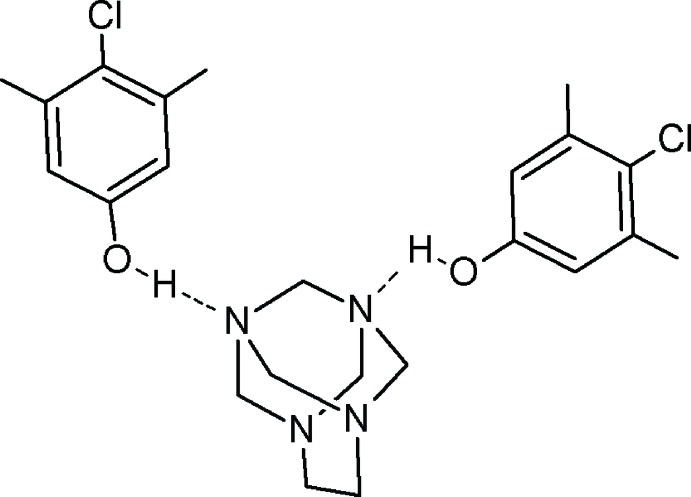


In recent years, we have become inter­ested in this cage aminal, which contains two pairs of non-equivalent nitro­gen atoms. Another intriguing feature of TATU is that, in contrast with the related aminal 1,3,6,8-tetra­aza­tri­cyclo­[4.4.1.1^3,8^]dodecane (TATD) for example (Riddell & Murray-Rust, 1970 ▸), TATU did not react with phenols when the reaction was attempted under standard conditions in various organic solvents. Instead, the reaction only took place when the mixture was at heated in an oil-bath at 393 K for 15 min under solvent-free conditions, affording symmetrical 1,3-bis­(2-hy­droxy­benz­yl)imidazolidines (BISBIAs) in good yields (Hernández, 2007 ▸). We also discovered that, under mechanochemical conditions, grinding the reagents in a mortar and pestle, the reaction of TATU with phenols affords phenol–aminal aggregates in excellent yields. Furthermore, no side products form in the reaction mixture. Usually, washing the homogeneous mixture with an appropriate solvent and filtration of the solid gives the pure adduct. In this article, we report the crystal structure of the title compound, an adduct obtained on milling a 1:2 stoichiometric mixture of TATU and 4-chloro-3,5-di­methyl­phenol in an agate mortar. This mechanochemical process provides a convenient and efficient method to produce these adducts, and is also environmentally friendly.

## Structural commentary   

**Figure 1:**
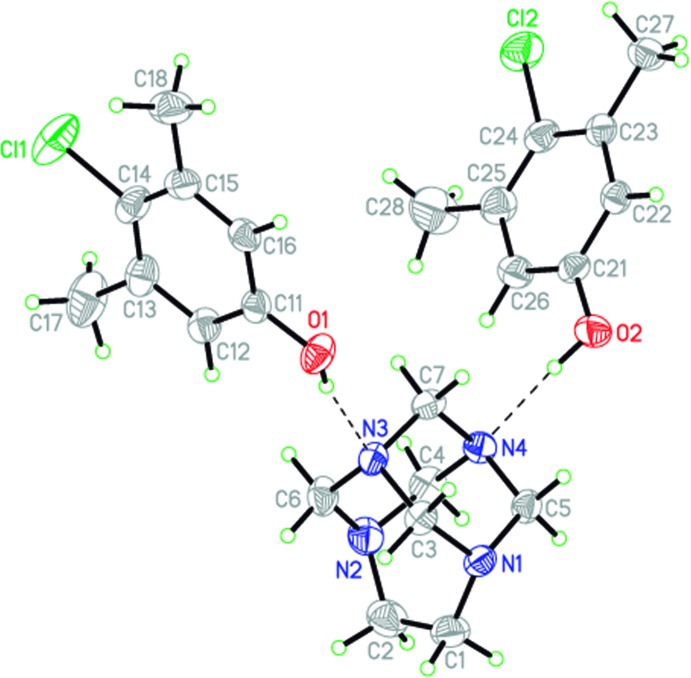
The mol­ecular structure of the title compound, with displacement ellipsoids drawn at the 50% probability level. Hydrogen bonds are shown as dashed lines.

The title compound crystallizes in space group *P2_1_/n* with one aminal cage mol­ecule and two 4-chloro-3,5-di­methyl­phenol mol­ecules in the asymmetric unit (Fig. 1 ▸) linked by two hydrogen bonds (Table 1 ▸). Nitro­gen atoms with the higher *sp* ^3^ character act as acceptors in this case, with Σα_(C–N–C)_ = 328.18 and 327.77° for N3 and N4, respectively, as seen with a previous reported TATU hydro­quinone adduct (Rivera *et al.*, 2007 ▸). The geometry of the N–C–C–N group of the adamanzane cage in the title compound is slightly distorted from a *syn* periplanar geometry, as evidenced by the N1—C1—C2—N2 dihedral angle [2.7 (3)°].

## Supra­molecular features   

**Figure 2:**
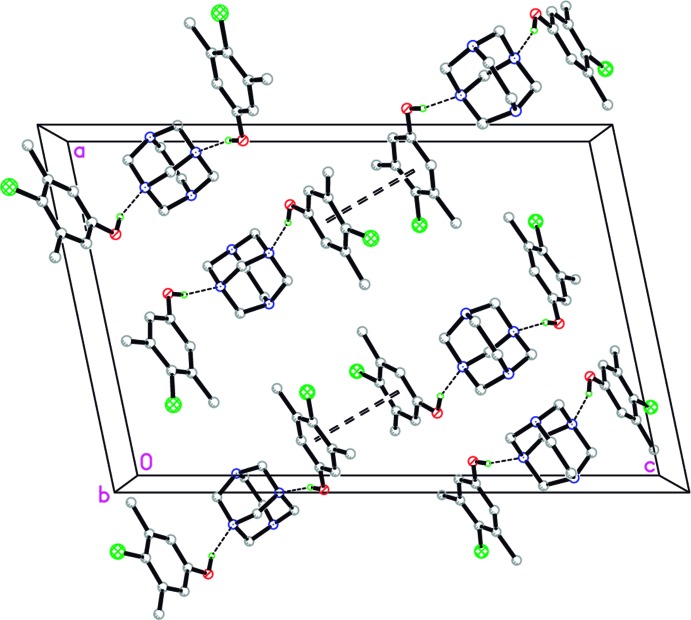
Packing diagram for title compound, viewed along the *b* axis.

In addition to the O—H⋯N contacts that form the 1:2 co-crystals, weak offset π–π stacking inter­actions link adjacent O1 and O2 phenol rings with a rather long separation between the centroids [*Cg*8⋯*Cg*9^i^ = 4.0570 (11); symmetry code: (i) 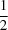 + *x*, 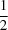 − *y*, 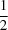 + *z; *Cg**8 and *Cg*9 are the centroids of the C11–16 and C21–C26 rings, respectively] and the benzene ring planes are inclined to one another by 0.58 (9)°. These additional contacts link the three-membered co-crystal units into chains approximately parallel to (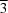
03), Fig. 2 ▸.

## Database survey   

Only three comparable structures were found in the Cambridge Structural Database (Groom *et al.* 2016 ▸), namely 1,3,6,8-tetra-aza­tri­cyclo­(4.3.1.1^3,8^)undecane hydro­quinone (HICTOD; Rivera *et al.*, 2007 ▸), 3,6,8-tri­aza-1-azoniatri­cyclo­[4.3.1.1^3,8^]undecane penta­chloro­phenolate monohydrate (OMODEA; Rivera *et al.*, 2011 ▸), and 4-nitro­phenol 1,3,6,8-tetra-aza­tri­cyclo­[4.3.1.1^3,8^]undecane (VUXMEI; Rivera *et al.*, 2015 ▸).

## Synthesis and crystallization   

A mixture of 1,3,6,8-tetra­aza­tri­cyclo­[4.3.1.1^3,8^]undecano (TATU) (154 mg, 1 mmol) and 4-chloro-3,5-di­methyl­phenol (313 mg, 2 mmol) was ground using a mortar and pestle at room temperature for 15 min. Completion of the reaction was monitored by TLC. The mixture was recrystallized from *n*-hexane solution to obtain colourless crystals suitable for X-ray analysis, m.p. = 375–376 K. (yield: 63%).

## Refinement   

Crystal data, data collection and structure refinement details are summarized in Table 2 ▸. All H atoms were located in a difference electron-density map. C-bound H atoms were fixed geometrically (C—H = 0.95 or 0.99Å) and refined using a riding-model approximation, with *U*_iso_(H) set to 1.2*U*_eq_ of the parent atom. The hydroxyl H atoms were refined freely.

## Supplementary Material

Crystal structure: contains datablock(s) I. DOI: 10.1107/S2056989016016650/sj5512sup1.cif

Structure factors: contains datablock(s) I. DOI: 10.1107/S2056989016016650/sj5512Isup2.hkl

CCDC reference: 1510356

Additional supporting information: crystallographic information; 3D view; checkCIF report

## Figures and Tables

**Table 1:** Hydrogen-bond geometry (Å, °)

*D*—H⋯*A*	*D*—H	H⋯*A*	*D*⋯*A*	*D*—H⋯*A*
O1—H1⋯N3	0.82 (3)	1.96 (3)	2.766 (2)	166 (3)
O2—H2⋯N4	0.89 (3)	1.90 (3)	2.760 (2)	160 (2)

**Table 2:** Experimental details

Crystal data	
Chemical formula	C_7_H_14_N_4_·2C_8_H_9_ClO
*M*_r_	467.42
Crystal system, space group	Monoclinic, *P*2_1_/*n*
Temperature (K)	173
*a*, *b*, *c* (Å)	14.5170 (8), 7.6178 (4), 22.1756 (11)
β (°)	101.824 (4)
*V* (Å^3^)	2400.3 (2)
*Z*	4
Radiation type	Mo *K*α
μ (mm^−1^)	0.30
Crystal size (mm)	0.28 × 0.24 × 0.24

Data collection	
Diffractometer	STOE IPDS II two-circle
Absorption correction	Multi-scan (*X-AREA*; Stoe & Cie, 2001 ▸)
*T*_min_, *T*_max_	0.609, 1.000
No. of measured, independent and observed [*I* > 2σ(*I*)] reflections	23030, 4501, 3584
*R*_int_	0.032
(sin θ/λ)_max_ (Å^−1^)	0.611

Refinement	
*R*[*F* ^2^ > 2σ(*F* ^2^)], *wR*(*F* ^2^), *S*	0.042, 0.100, 1.03
No. of reflections	4501
No. of parameters	292
H-atom treatment	H atoms treated by a mixture of independent and constrained refinement
Δρ_max_, Δρ_min_ (e Å^−3^)	0.28, −0.30

Computer programs: *X-AREA* (Stoe & Cie, 2001 ▸), *SHELXS* (Sheldrick, 2008 ▸), *SHELXL2014/7* (Sheldrick, 2015 ▸) and *XP* in *SHELXTL-Plus* (Sheldrick, 2008 ▸).

## supplementary crystallographic information

### Crystal data

**Table:**

C_7_H_14_N_4_·2C_8_H_9_ClO	*F*(000) = 992
*M**_r_* = 467.42	*D*_x_ = 1.293 Mg m^−^^3^
Monoclinic, *P*2_1_/*n*	Mo *K*α radiation, λ = 0.71073 Å
*a* = 14.5170 (8) Å	Cell parameters from 23030 reflections
*b* = 7.6178 (4) Å	θ = 3.3–25.9°
*c* = 22.1756 (11) Å	µ = 0.30 mm^−^^1^
β = 101.824 (4)°	*T* = 173 K
*V* = 2400.3 (2) Å^3^	Block, colourless
*Z* = 4	0.28 × 0.24 × 0.24 mm

### Data collection

**Table:**

STOE IPDS II two-circle diffractometer	3584 reflections with *I* > 2σ(*I*)
Radiation source: Genix 3D IµS microfocus X-ray source	*R*_int_ = 0.032
ω scans	θ_max_ = 25.7°, θ_min_ = 3.3°
Absorption correction: multi-scan (X-Area; Stoe & Cie, 2001)	*h* = −17→17
*T*_min_ = 0.609, *T*_max_ = 1.000	*k* = −9→9
23030 measured reflections	*l* = −26→26
4501 independent reflections	

### Refinement

**Table:**

Refinement on *F*^2^	0 restraints
Least-squares matrix: full	Hydrogen site location: mixed
*R*[*F*^2^ > 2σ(*F*^2^)] = 0.042	H atoms treated by a mixture of independent and constrained refinement
*wR*(*F*^2^) = 0.100	*w* = 1/[σ^2^(*F*_o_^2^) + (0.0488*P*)^2^ + 0.6672*P*] where *P* = (*F*_o_^2^ + 2*F*_c_^2^)/3
*S* = 1.03	(Δ/σ)_max_ = 0.001
4501 reflections	Δρ_max_ = 0.28 e Å^−^^3^
292 parameters	Δρ_min_ = −0.30 e Å^−^^3^

### Special details

**Table:**

Geometry. All esds (except the esd in the dihedral angle between two l.s. planes) are estimated using the full covariance matrix. The cell esds are taken into account individually in the estimation of esds in distances, angles and torsion angles; correlations between esds in cell parameters are only used when they are defined by crystal symmetry. An approximate (isotropic) treatment of cell esds is used for estimating esds involving l.s. planes.

### Fractional atomic coordinates and isotropic or equivalent isotropic displacement parameters (Å^2^)

**Table:**

	*x*	*y*	*z*	*U*_iso_*/*U*_eq_	
N1	0.32321 (11)	1.00388 (19)	0.72849 (7)	0.0303 (3)	
N2	0.48631 (12)	0.9345 (2)	0.67795 (8)	0.0386 (4)	
N3	0.43541 (11)	0.75773 (19)	0.75911 (7)	0.0287 (3)	
N4	0.33805 (11)	0.76332 (19)	0.65546 (7)	0.0291 (3)	
C1	0.37625 (16)	1.1541 (3)	0.71281 (10)	0.0417 (5)	
H1A	0.3950	1.2268	0.7503	0.050*	
H1B	0.3331	1.2259	0.6821	0.050*	
C2	0.46513 (17)	1.1172 (3)	0.68706 (11)	0.0488 (5)	
H2A	0.4585	1.1784	0.6470	0.059*	
H2B	0.5197	1.1697	0.7155	0.059*	
C3	0.37388 (13)	0.8935 (2)	0.77837 (8)	0.0305 (4)	
H3A	0.3274	0.8343	0.7984	0.037*	
H3B	0.4130	0.9700	0.8096	0.037*	
C4	0.41772 (15)	0.8403 (3)	0.63247 (9)	0.0373 (5)	
H4A	0.4506	0.7448	0.6152	0.045*	
H4B	0.3922	0.9219	0.5984	0.045*	
C5	0.27736 (14)	0.8983 (2)	0.67616 (8)	0.0310 (4)	
H5A	0.2533	0.9778	0.6412	0.037*	
H5B	0.2224	0.8387	0.6871	0.037*	
C6	0.51400 (14)	0.8331 (3)	0.73454 (9)	0.0368 (4)	
H6A	0.5514	0.9097	0.7665	0.044*	
H6B	0.5555	0.7361	0.7268	0.044*	
C7	0.37716 (14)	0.6537 (2)	0.70954 (8)	0.0307 (4)	
H7A	0.3249	0.5985	0.7252	0.037*	
H7B	0.4159	0.5588	0.6970	0.037*	
Cl1	0.76106 (5)	−0.00986 (9)	0.86534 (3)	0.0680 (2)	
O1	0.45719 (10)	0.5078 (2)	0.85145 (7)	0.0395 (3)	
H1	0.461 (2)	0.582 (4)	0.8252 (13)	0.066 (9)*	
C11	0.52937 (13)	0.3920 (2)	0.85398 (8)	0.0299 (4)	
C12	0.60769 (14)	0.4285 (3)	0.82904 (8)	0.0340 (4)	
H12	0.6118	0.5383	0.8094	0.041*	
C13	0.68020 (15)	0.3070 (3)	0.83229 (9)	0.0394 (5)	
C14	0.67140 (15)	0.1475 (3)	0.86136 (9)	0.0391 (5)	
C15	0.59503 (15)	0.1077 (2)	0.88797 (8)	0.0369 (5)	
C16	0.52400 (14)	0.2321 (2)	0.88326 (8)	0.0328 (4)	
H16	0.4706	0.2073	0.9004	0.039*	
C17	0.76413 (18)	0.3517 (4)	0.80495 (12)	0.0633 (7)	
H17A	0.7570	0.4708	0.7879	0.095*	
H17B	0.7685	0.2680	0.7721	0.095*	
H17C	0.8215	0.3453	0.8371	0.095*	
C18	0.58762 (19)	−0.0650 (3)	0.92064 (11)	0.0518 (6)	
H18A	0.5329	−0.0619	0.9401	0.078*	
H18B	0.6448	−0.0835	0.9522	0.078*	
H18C	0.5804	−0.1612	0.8907	0.078*	
Cl2	0.31094 (4)	−0.07547 (7)	0.46192 (3)	0.05106 (17)	
O2	0.20265 (11)	0.53522 (18)	0.59453 (7)	0.0378 (3)	
H2	0.252 (2)	0.606 (4)	0.6060 (12)	0.057 (7)*	
C21	0.23133 (14)	0.3965 (2)	0.56384 (8)	0.0295 (4)	
C22	0.17280 (14)	0.2507 (2)	0.55338 (8)	0.0297 (4)	
H22	0.1157	0.2507	0.5681	0.036*	
C23	0.19606 (13)	0.1043 (2)	0.52177 (8)	0.0298 (4)	
C24	0.28058 (15)	0.1093 (2)	0.50121 (8)	0.0343 (4)	
C25	0.34103 (15)	0.2525 (3)	0.51093 (10)	0.0406 (5)	
C26	0.31481 (15)	0.3973 (3)	0.54264 (9)	0.0365 (4)	
H26	0.3547	0.4972	0.5497	0.044*	
C27	0.13190 (16)	−0.0528 (3)	0.51162 (9)	0.0389 (5)	
H27A	0.1059	−0.0664	0.4675	0.058*	
H27B	0.0804	−0.0360	0.5336	0.058*	
H27C	0.1676	−0.1583	0.5272	0.058*	
C28	0.4326 (2)	0.2544 (4)	0.48876 (15)	0.0711 (8)	
H28A	0.4759	0.1682	0.5121	0.107*	
H28B	0.4608	0.3716	0.4949	0.107*	
H28C	0.4207	0.2245	0.4449	0.107*	

### Atomic displacement parameters (Å^2^)

**Table:**

	*U*^11^	*U*^22^	*U*^33^	*U*^12^	*U*^13^	*U*^23^
N1	0.0329 (9)	0.0276 (8)	0.0293 (8)	0.0056 (7)	0.0039 (7)	−0.0013 (6)
N2	0.0354 (9)	0.0409 (9)	0.0402 (9)	−0.0042 (8)	0.0095 (8)	0.0104 (7)
N3	0.0250 (8)	0.0308 (8)	0.0310 (8)	0.0028 (6)	0.0070 (6)	0.0059 (6)
N4	0.0319 (9)	0.0288 (8)	0.0276 (7)	−0.0004 (7)	0.0082 (7)	−0.0025 (6)
C1	0.0506 (13)	0.0288 (10)	0.0423 (11)	−0.0006 (9)	0.0015 (10)	0.0027 (8)
C2	0.0509 (14)	0.0415 (12)	0.0519 (13)	−0.0107 (10)	0.0053 (11)	0.0087 (10)
C3	0.0334 (10)	0.0332 (9)	0.0246 (8)	0.0036 (8)	0.0053 (8)	−0.0007 (7)
C4	0.0407 (12)	0.0431 (11)	0.0309 (10)	0.0008 (9)	0.0138 (9)	0.0035 (8)
C5	0.0296 (10)	0.0330 (9)	0.0285 (9)	0.0039 (8)	0.0014 (7)	−0.0018 (8)
C6	0.0267 (10)	0.0452 (11)	0.0386 (10)	−0.0007 (9)	0.0068 (8)	0.0098 (9)
C7	0.0340 (10)	0.0249 (8)	0.0354 (10)	0.0029 (8)	0.0118 (8)	0.0021 (7)
Cl1	0.0767 (5)	0.0679 (4)	0.0573 (4)	0.0424 (4)	0.0085 (3)	−0.0006 (3)
O1	0.0345 (8)	0.0433 (8)	0.0426 (8)	0.0090 (6)	0.0122 (6)	0.0165 (7)
C11	0.0282 (10)	0.0333 (9)	0.0262 (8)	0.0007 (8)	0.0011 (7)	0.0004 (7)
C12	0.0347 (11)	0.0367 (10)	0.0299 (9)	0.0003 (8)	0.0049 (8)	0.0060 (8)
C13	0.0359 (11)	0.0503 (12)	0.0315 (10)	0.0066 (9)	0.0056 (9)	0.0006 (9)
C14	0.0438 (12)	0.0394 (11)	0.0306 (10)	0.0132 (9)	−0.0010 (9)	−0.0045 (8)
C15	0.0481 (12)	0.0283 (10)	0.0275 (9)	−0.0005 (9)	−0.0080 (9)	−0.0018 (7)
C16	0.0339 (11)	0.0343 (10)	0.0278 (9)	−0.0065 (8)	0.0004 (8)	0.0022 (8)
C17	0.0464 (15)	0.0883 (19)	0.0611 (15)	0.0167 (14)	0.0248 (13)	0.0160 (14)
C18	0.0695 (17)	0.0326 (11)	0.0457 (12)	−0.0027 (11)	−0.0063 (11)	0.0054 (9)
Cl2	0.0568 (4)	0.0446 (3)	0.0520 (3)	0.0121 (3)	0.0117 (3)	−0.0150 (2)
O2	0.0397 (8)	0.0314 (7)	0.0422 (8)	−0.0010 (6)	0.0079 (6)	−0.0103 (6)
C21	0.0357 (10)	0.0258 (9)	0.0254 (8)	0.0051 (8)	0.0021 (8)	0.0006 (7)
C22	0.0297 (10)	0.0326 (9)	0.0258 (9)	0.0019 (8)	0.0033 (7)	0.0006 (7)
C23	0.0343 (10)	0.0297 (9)	0.0225 (8)	0.0027 (8)	−0.0009 (7)	0.0019 (7)
C24	0.0402 (11)	0.0313 (10)	0.0303 (9)	0.0075 (8)	0.0048 (8)	−0.0029 (8)
C25	0.0386 (12)	0.0407 (11)	0.0447 (11)	0.0030 (9)	0.0139 (9)	−0.0002 (9)
C26	0.0362 (11)	0.0319 (10)	0.0418 (11)	−0.0041 (8)	0.0091 (9)	−0.0004 (8)
C27	0.0461 (12)	0.0317 (10)	0.0360 (10)	−0.0050 (9)	0.0017 (9)	−0.0023 (8)
C28	0.0551 (17)	0.0679 (17)	0.102 (2)	−0.0075 (14)	0.0432 (16)	−0.0208 (16)

### Geometric parameters (Å, º)

**Table:**

N1—C5	1.456 (2)	C12—H12	0.9500
N1—C1	1.460 (3)	C13—C14	1.393 (3)
N1—C3	1.462 (2)	C13—C17	1.507 (3)
N2—C2	1.448 (3)	C14—C15	1.392 (3)
N2—C4	1.453 (3)	C15—C16	1.388 (3)
N2—C6	1.458 (2)	C15—C18	1.516 (3)
N3—C7	1.473 (2)	C16—H16	0.9500
N3—C6	1.477 (2)	C17—H17A	0.9800
N3—C3	1.485 (2)	C17—H17B	0.9800
N4—C7	1.476 (2)	C17—H17C	0.9800
N4—C4	1.478 (2)	C18—H18A	0.9800
N4—C5	1.487 (2)	C18—H18B	0.9800
C1—C2	1.540 (3)	C18—H18C	0.9800
C1—H1A	0.9900	Cl2—C24	1.7585 (19)
C1—H1B	0.9900	O2—C21	1.367 (2)
C2—H2A	0.9900	O2—H2	0.89 (3)
C2—H2B	0.9900	C21—C26	1.387 (3)
C3—H3A	0.9900	C21—C22	1.389 (3)
C3—H3B	0.9900	C22—C23	1.395 (3)
C4—H4A	0.9900	C22—H22	0.9500
C4—H4B	0.9900	C23—C24	1.394 (3)
C5—H5A	0.9900	C23—C27	1.505 (3)
C5—H5B	0.9900	C24—C25	1.389 (3)
C6—H6A	0.9900	C25—C26	1.402 (3)
C6—H6B	0.9900	C25—C28	1.509 (3)
C7—H7A	0.9900	C26—H26	0.9500
C7—H7B	0.9900	C27—H27A	0.9800
Cl1—C14	1.759 (2)	C27—H27B	0.9800
O1—C11	1.362 (2)	C27—H27C	0.9800
O1—H1	0.82 (3)	C28—H28A	0.9800
C11—C16	1.390 (3)	C28—H28B	0.9800
C11—C12	1.390 (3)	C28—H28C	0.9800
C12—C13	1.392 (3)		
			
C5—N1—C1	114.85 (15)	C11—C12—C13	121.18 (18)
C5—N1—C3	111.23 (14)	C11—C12—H12	119.4
C1—N1—C3	115.00 (16)	C13—C12—H12	119.4
C2—N2—C4	115.88 (18)	C12—C13—C14	117.61 (18)
C2—N2—C6	114.72 (17)	C12—C13—C17	119.6 (2)
C4—N2—C6	111.36 (16)	C14—C13—C17	122.8 (2)
C7—N3—C6	107.59 (14)	C15—C14—C13	122.87 (18)
C7—N3—C3	107.58 (14)	C15—C14—Cl1	118.40 (16)
C6—N3—C3	113.01 (15)	C13—C14—Cl1	118.73 (16)
C7—N4—C4	107.88 (15)	C16—C15—C14	117.60 (18)
C7—N4—C5	107.10 (13)	C16—C15—C18	120.2 (2)
C4—N4—C5	112.79 (15)	C14—C15—C18	122.2 (2)
N1—C1—C2	117.90 (16)	C15—C16—C11	121.41 (18)
N1—C1—H1A	107.8	C15—C16—H16	119.3
C2—C1—H1A	107.8	C11—C16—H16	119.3
N1—C1—H1B	107.8	C13—C17—H17A	109.5
C2—C1—H1B	107.8	C13—C17—H17B	109.5
H1A—C1—H1B	107.2	H17A—C17—H17B	109.5
N2—C2—C1	116.43 (17)	C13—C17—H17C	109.5
N2—C2—H2A	108.2	H17A—C17—H17C	109.5
C1—C2—H2A	108.2	H17B—C17—H17C	109.5
N2—C2—H2B	108.2	C15—C18—H18A	109.5
C1—C2—H2B	108.2	C15—C18—H18B	109.5
H2A—C2—H2B	107.3	H18A—C18—H18B	109.5
N1—C3—N3	114.96 (14)	C15—C18—H18C	109.5
N1—C3—H3A	108.5	H18A—C18—H18C	109.5
N3—C3—H3A	108.5	H18B—C18—H18C	109.5
N1—C3—H3B	108.5	C21—O2—H2	107.7 (17)
N3—C3—H3B	108.5	O2—C21—C26	122.80 (17)
H3A—C3—H3B	107.5	O2—C21—C22	117.58 (17)
N2—C4—N4	115.46 (15)	C26—C21—C22	119.62 (16)
N2—C4—H4A	108.4	C21—C22—C23	121.36 (17)
N4—C4—H4A	108.4	C21—C22—H22	119.3
N2—C4—H4B	108.4	C23—C22—H22	119.3
N4—C4—H4B	108.4	C24—C23—C22	117.53 (17)
H4A—C4—H4B	107.5	C24—C23—C27	122.14 (17)
N1—C5—N4	115.16 (15)	C22—C23—C27	120.33 (17)
N1—C5—H5A	108.5	C25—C24—C23	122.76 (17)
N4—C5—H5A	108.5	C25—C24—Cl2	119.41 (15)
N1—C5—H5B	108.5	C23—C24—Cl2	117.82 (15)
N4—C5—H5B	108.5	C24—C25—C26	117.89 (18)
H5A—C5—H5B	107.5	C24—C25—C28	121.85 (19)
N2—C6—N3	115.20 (16)	C26—C25—C28	120.3 (2)
N2—C6—H6A	108.5	C21—C26—C25	120.83 (18)
N3—C6—H6A	108.5	C21—C26—H26	119.6
N2—C6—H6B	108.5	C25—C26—H26	119.6
N3—C6—H6B	108.5	C23—C27—H27A	109.5
H6A—C6—H6B	107.5	C23—C27—H27B	109.5
N3—C7—N4	111.60 (14)	H27A—C27—H27B	109.5
N3—C7—H7A	109.3	C23—C27—H27C	109.5
N4—C7—H7A	109.3	H27A—C27—H27C	109.5
N3—C7—H7B	109.3	H27B—C27—H27C	109.5
N4—C7—H7B	109.3	C25—C28—H28A	109.5
H7A—C7—H7B	108.0	C25—C28—H28B	109.5
C11—O1—H1	109 (2)	H28A—C28—H28B	109.5
O1—C11—C16	118.06 (17)	C25—C28—H28C	109.5
O1—C11—C12	122.63 (17)	H28A—C28—H28C	109.5
C16—C11—C12	119.31 (18)	H28B—C28—H28C	109.5
			
C5—N1—C1—C2	−67.1 (2)	C12—C13—C14—C15	−1.4 (3)
C3—N1—C1—C2	63.9 (2)	C17—C13—C14—C15	178.4 (2)
C4—N2—C2—C1	64.0 (2)	C12—C13—C14—Cl1	179.18 (15)
C6—N2—C2—C1	−68.0 (3)	C17—C13—C14—Cl1	−1.0 (3)
N1—C1—C2—N2	2.7 (3)	C13—C14—C15—C16	1.9 (3)
C5—N1—C3—N3	48.2 (2)	Cl1—C14—C15—C16	−178.69 (14)
C1—N1—C3—N3	−84.51 (19)	C13—C14—C15—C18	−178.64 (19)
C7—N3—C3—N1	−54.22 (19)	Cl1—C14—C15—C18	0.7 (3)
C6—N3—C3—N1	64.4 (2)	C14—C15—C16—C11	−1.1 (3)
C2—N2—C4—N4	−85.9 (2)	C18—C15—C16—C11	179.49 (18)
C6—N2—C4—N4	47.6 (2)	O1—C11—C16—C15	−179.65 (17)
C7—N4—C4—N2	−53.5 (2)	C12—C11—C16—C15	−0.2 (3)
C5—N4—C4—N2	64.6 (2)	O2—C21—C22—C23	179.19 (16)
C1—N1—C5—N4	84.1 (2)	C26—C21—C22—C23	−0.1 (3)
C3—N1—C5—N4	−48.8 (2)	C21—C22—C23—C24	0.2 (3)
C7—N4—C5—N1	54.95 (19)	C21—C22—C23—C27	179.35 (17)
C4—N4—C5—N1	−63.6 (2)	C22—C23—C24—C25	−0.1 (3)
C2—N2—C6—N3	85.9 (2)	C27—C23—C24—C25	−179.16 (19)
C4—N2—C6—N3	−48.2 (2)	C22—C23—C24—Cl2	179.44 (13)
C7—N3—C6—N2	54.5 (2)	C27—C23—C24—Cl2	0.4 (2)
C3—N3—C6—N2	−64.1 (2)	C23—C24—C25—C26	−0.2 (3)
C6—N3—C7—N4	−60.74 (18)	Cl2—C24—C25—C26	−179.73 (15)
C3—N3—C7—N4	61.31 (17)	C23—C24—C25—C28	179.4 (2)
C4—N4—C7—N3	60.23 (18)	Cl2—C24—C25—C28	−0.1 (3)
C5—N4—C7—N3	−61.44 (18)	O2—C21—C26—C25	−179.47 (18)
O1—C11—C12—C13	−179.85 (18)	C22—C21—C26—C25	−0.2 (3)
C16—C11—C12—C13	0.7 (3)	C24—C25—C26—C21	0.4 (3)
C11—C12—C13—C14	0.1 (3)	C28—C25—C26—C21	−179.3 (2)
C11—C12—C13—C17	−179.7 (2)		

### Hydrogen-bond geometry (Å, º)

**Table:**

*D*—H···*A*	*D*—H	H···*A*	*D*···*A*	*D*—H···*A*
O1—H1···N3	0.82 (3)	1.96 (3)	2.766 (2)	166 (3)
O2—H2···N4	0.89 (3)	1.90 (3)	2.760 (2)	160 (2)
